# Glomerular IgA Deposition and Serum Antineutrophil Cytoplasmic Antibody Positivity in a Child With Dystrophic Epidermolysis Bullosa: Case Report and Literature Review

**DOI:** 10.3389/fped.2022.939069

**Published:** 2022-07-11

**Authors:** Ling Yu, Guoping Huang, Zhihong Lu, Jingjing Wang, Weizhong Gu, Junping Li, Jianhua Mao

**Affiliations:** ^1^Department of Nephrology, The Children’s Hospital, Zhejiang University School of Medicine, National Clinical Research Center for Child Health, Hangzhou, China; ^2^Department of Pathology, The Children’s Hospital, Zhejiang University School of Medicine, National Clinical Research Center for Child Health, Hangzhou, China

**Keywords:** dystrophic epidermolysis bullosa, IgA nephropathy, antineutrophil cytoplasmic antibodies, anti-MPO, anti-PR3, child

## Abstract

Patients with epidermolysis bullosa (EB) could develop significant urological complications, such as hydroureteronephrosis, renal amyloidosis and IgA nephropathy (IgAN). Here, we presented a 12-year-old boy carrying pathogenic COL7A1 mutation with diagnosis of dystrophic epidermolysis bullosa (DEB). The patient had concomitant gross hematuria and proteinuria. Pathological examinations and immunostaining of renal biopsy showed glomeruli with mesangial hypercellularity and deposition of IgA, which were indicative of IgAN. Interestingly, serological evaluation showed antineutrophil cytoplasmic antibody (ANCA) directed against myeloperoxidase and proteinase 3. Treatment with glucocorticoid, immunosuppressants, angiotensin-converting enzyme inhibitor and antibiotics efficiently improved hemato-proteinuria, and ANCAs became negative as well. This case of DEB presented a unique collection of clinical manifestations and pathological alterations. IgAN and serum positive ANCA were possibly associated with sustained infection secondary to DEB, and can be managed by empirical treatment for primary IgAN.

## Introduction

Dystrophic epidermolysis bullosa (DEB) is a monogenetic disease with clinical manifestations of painful and easy-blistering skin and mucous membranes secondary to friction or minor trauma (1). The lesions leave erosions and scars that, in turn, can cause stenosis of tracheal, esophageal, and genitourinary tract mucosae (2). DEB is caused by mutations in COL7A1, the gene encoding type VII collagen, a major protein component of the anchoring fibrils that play a critical role in securing the attachment of the dermal-epidermal basement membrane to the underlying dermis (3). A proportion of patients with DEB had urological complications, such as hydroureteronephrosis, renal amyloidosis, and IgA nephropathy (IgAN) (1, 4, 5).

IgAN is the most common form of glomerulonephritis and is characterized by the predominance of IgA deposits either alone or with other immune deposits in the glomerular mesangium. To date, the pathogenesis of IgAN remains undefined (6). ANCA-associated vasculitis (AAV) is an autoimmune disorder involving severe, systemic and small-vessel vasculitis in multiple organs. In kidneys affected by AAV, the characteristic lesion is segmental necrosis of glomerular capillary loops, with little or no deposition of immunoglobulin or complement, and termed as “pauci-immune” focal necrotizing glomerulonephritis (7). ANCAs are autoantibodies directed against cytoplasmic constituents of neutrophils. Based on their appearance on indirect immunofluorescence microscopy, ANCAs are classified as perinuclear (P-ANCA) or cytoplasmic (C-ANCA) (8). Their most common antigens have been identified as myeloperoxidase (MPO) and proteinase 3 (PR3), respectively (9, 10). Antibody against MPO (anti-MPO) and PR3 (anti-PR3) are used as diagnostic markers for AAV (7). Interestingly, a number of clinical observations showed ANCAs are not only exclusively for AAV, but can also be detected in other diseases, such as systemic lupus erythematosus (SLE), inflammatory bowel disease, malignancy, drug-induced AAV, infections, and IgAN (11–13). The significance of serum ANCA positivity in patients without AAV remains largely undefined.

Concomitant presentation of IgAN and AAV is rarely reported (12). Here we describe for the first time a case of pediatric DEB with serum anti-MPO and anti-PR3 positivity, accompanied by histological evidence of IgA nephropathy.

## Case Report

**FIGURE 1:**
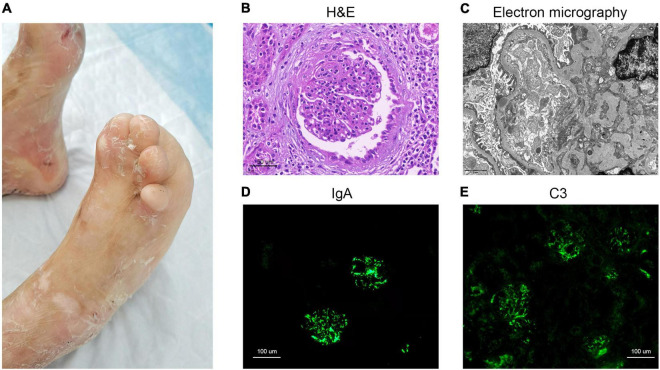
Clinical image of the dystrophic epidermolysis bullosa patient and histology from renal biopsy. **(A)** Skin scarring on feet, pseudosyndactyly and nail dystrophy. **(B)** H&E staining. **(C)** Electron micrography image. **(D)** IgA immunofluorescence staining. **(E)** C3 immunofluorescence staining.

A 12-year-old boy was diagnosed at birth with EB characterized by recurrent blisters and erosions on skin and oral mucosa. The patient had limited mouth opening, irregular dentition, nail dystrophy, pseudosyndactyly, and flexion deformities of interphalangeal joints due to progressive scarring of the extremities (Figure 1A). Besides, he suffered from gross hematuria and proteinuria. He had previously normal urinalysis result when he was young, but it was not monitored regularly. He visited the outpatient clinic of our hospital immediately after the appearance of gross hematuria in January 2021, and urine routine test also revealed massive proteinuria. But the patient had no significant hypoproteinemia, edema, and hyperlipidemia. More information is shown in Table 1.

**TABLE 1:** Laboratory data on first admission.

	Results	Reference range
**Urine**		
Occult blood	3 +	Negative
RBCs (per HPF)	322	0–3
Protein/creatinine ratio (mg/mg)	3.49	<0.20
24-h urinary protein (mg)	1273.3	<150
**Blood**		
WBC (thousand/mm^3^)	5.69	4.00–12.00
Hemoglobin (g/L)	75	110–155
Platelet (thousand/mm^3^)	328	100–400
hsCRP (mg/L)	13.25	0.00–8.00
SAA (mg/L)	32.2	0.0–10.0
Albumin (g/L)	28.1	32.0–52.0
Cholesterol (mmol/L)	4.01	3.00–5.70
Urea nitrogen (mmol/L)	2.10	2.80–7.60
Creatinine (μmol/L)	25	21–65
ESR (mm/h)	>110	0–20
ANA	1:100	<1:100
Anti-dsDNA (IU/mL)	<1:100	<1:100
P-ANCA	1:100	<1:20
C-ANCA	1:100	<1:20
Anti-MPO (RU/mL)	132.20	0–20
Anti-PR3 (RU/mL)	71.11	0–20
Immunoglobulin G (g/L)	23.8	6.36–13.24
Immunoglobulin A (g/L)	9.10	0.49–2.29
Immunoglobulin M (g/L)	1.99	0.42–1.46
Immunoglobulin E (IU/mL)	315.0	0.0–100.0
C3 (g/L)	1.602	0.900–1.800
C4 (g/L)	0.347	0.100–0.400
Viral hepatitis panel	Negative	Negative
HIV, syphilis and TB screening	Negative	Negative

*RBCs, red blood cells; WBC, white blood cells; hsCRP, hypersensitive C-reactive protein; SAA, serum amyloid A; ESR, erythrocyte sedimentation rate; ANA, anti-nuclear antibody; Anti-dsDNA, anti-double-stranded DNA antibody; P-ANCA, perinuclear antineutrophil cytoplasmic antibody; C-ANCA, cytoplasmic antineutrophil cytoplasmic antibody; Anti-MPO, antibody against myeloperoxidase; Anti-PR3, antibody against proteinase 3; C3, complement 3; C4, complement 4; TB, tuberculosis.*

The autoimmune serology demonstrated positive results for P-ANCA, C-ANCA, anti-MPO, and anti-PR3 (Table 1). Serum protein electrophoresis showed elevated levels of IgG, IgA, IgM, and IgE. Renal ultrasound suggested mild hydronephrosis of left kidney. Neurological, respiratory, cardiovascular, and hearing examinations were normal. Computed tomography (CT) imaging of chest and paranasal sinuses indicated no significant abnormality. In light of the above description, our patient lacked systemic manifestations of AAV, such as paranasal sinusitis, hemorrhagic alveolitis, and other granulomatous lesions.

The patient had no relevant family history. During the first hospitalization, whole-exon sequencing (WES) disclosed he carried the COL7A1 mutation of c.2992 + 2 (T>G) in exon IVS22 (also present in his mother) and c.8038G > A (p. Gly2680Ser) in exon 108 (also present in his father). His half-sister is healthy, but she has the same heterozygous mutation as his mother. Combined with clinical manifestation, he was diagnosed with autosomal recessive DEB (OMIM: 226600).

Pathological examination of renal biopsy showed glomeruli with mesangial hypercellularity and a diffuse increase of mesangial matrix. It revealed vacuolar and granular degeneration of tubular cells, partial atrophy of tubule, interstitial edema, and mild interstitial fibrosis with scattered clusters of lymphocyte and mononuclear cell infiltration in interstitium accompanied by vascular wall thickening of interstitial vessels (Figure 1B). Congo red staining was negative. The depositions of IgA, C3, and fibrinogen were observed in mesangial area upon immunofluorescence (IF) staining (Figures 1D,E). Staining for IgG, IgM, C4, and C1q were all negative. Electron micrography (EM) showed proliferation of endothelial cells and segmental fusion of foot processes. There was no obvious thickening of glomerular basement membranes. High density electron dense deposits were observed in mesangium, and occasionally found in subepithelium and subendothelial lesions (Figure 1C). No abnormal deposition of monoclonal light chain was detected by immunoelectron microscopy.

In addition, the culture of pharyngeal swab, urine, and blood were all negative. Bacterial culture of fresh skin bullae from his left lower limb was positive for methicillin-resistant Staphylococcus aureus (MRSA). After previous oral cefixime, he changed to oral cotrimoxazole for anti-infection based on the result of wound culture susceptibility.

The patient was initially treated with oral prednisone (1 mg/kg⋅d), followed by a reducing course over subsequent months. From the third month after oral prednisone, oral Mycophenolate mofetil (MMF, 20 mg/kg⋅d) was added. As his renal function was preserved, we opted for non-specific antiproteinuric therapy with an oral inhibitor of renin-angiotensin-aldosterone system (enalapril) throughout the treatment course. Besides, the patient had moderate normocytic hypochromic anemia at initial presentation. The severe forms of EB are usually complicated by anemia which is caused by the combination of iron deficiency and chronic inflammation. Therefore, we gave him oral iron supplementation (iron proteinsuccinylate) and three doses of intravenous bolus of recombinant human erythropoietin (3000IU per dose).

**FIGURE 2:**
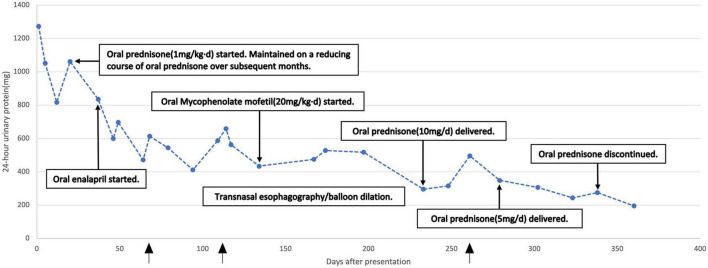
Twenty four hour urinary protein over the treatment course. The arrow indicated the time points when skin lesions worsened.

Gross hematuria disappeared 1 week after the patient started oral prednisone, and 1 month after it, proteinuria decreased to about one-half of that at admission. Twelve months after initiation of therapy, urinary occult blood became negative. 24-h urinary protein decreased to 195.5 mg (Figure 2). But whenever skin or mucosal infection occurred, urinary protein quantification transiently rises slightly than the previous measurement. After the wound recovered, it will decrease spontaneously. Then, the anemia improved markedly and hemoglobin increased to 110 g/L or more. Serum IgG and IgM fell into normal range, while IgA and IgE significantly decreased and were close to normal. Moreover, all relevant parameters of serum ANCA (P-ANCA, C-ANCA, anti-MPO, and anti-PR3) turned negative. Since January 2022, the child stopped oral prednisone, but continued to take MMF at the original dose. Due to the improvement of skin and mucosal infection, oral cotrimoxazole was discontinued.

## Discussion

The coexistence of IgAN with serum ANCA positivity was rarely reported. Notably, ANCA-positive IgAN all in a child with DEB has never been described. The causal relationship between the two entities is unclear. If IgAN overlaps with AAV, most patients present with rapidly progressive glomerulonephritis and necrotizing crescentic lesions (12, 14). They also exhibit systemic symptoms of vasculitis, including fever, hemoptysis, and arthralgia. But our case preserved renal function without these findings. It is possible that the patient had pre-existing IgAN, and positive anti-MPO and anti-PR3 results were an early finding of subsequent AAV. These ANCA titers would decrease to normal levels after immunosuppressive therapy. On the other hand, it could be mere incidental and non-pathogenic. Huang et al. reported a series of 14 patients from a total of 787 biopsied cases diagnosed of IgAN with ANCA seropositivity (15). Only three had crescentic glomerulonephritis. Of the remaining 11 patients without crescents, six had improved renal function. This finding may suggest that ANCA is not directly linked to crescent formation in IgAN.

Staphylococcus infection-associated glomerulonephritis (SAGN) develops in patients with chronic infection including superficial infection. Blood cultures might be negative (16). SAGN is common in the elderly, diabetic or immunodeficient patients, but rarely reported among children. Most cases were secondary to methicillin-resistant Staphylococcus aureus (MRSA) (17). The correlation between MRSA infection and glomerulonephritis has been well documented (6, 17). SAGN is characterized by glomerular IgA deposits, and it has also been termed IgA-dominant infection-associated glomerulonephritis (IAGN) (17, 18). Staphylococcal enterotoxins may act as superantigens which during inflammatory cascades promote proliferation and massive activation of T cells, production of proinflammatory cytokines and secretion of IgA, with circulating immune complexes formation and deposition in glomeruli. Codominant staining of glomerular IgA and Complement 3 (C3) is an important diagnostic feature of IAGN. Interestingly, some IAGN cases tested positive for ANCAs (16, 17). It is possible that infections may trigger ANCA formation through autoantigen complementarity, molecular mimicry, epigenetics, neutrophil extracellular traps (NETs) or Toll-like receptors (TLRs) (7).

As the incidence of MRSA infection increases globally, it could have emerged as the common cause of infection-associated glomerulonephritis in EB patients. Thus, we speculate that DEB patients may be diagnosed with SAGN or SAGN may superimpose on IgAN. However, EB cases with SAGN have rarely been reported. Due to complexity of DEB, differential diagnosis and accurate treatment seems to be a clinical problem. SAGN would improve after resolution of infection, but it may be reasonable to reserve immunosuppressive therapy for patients with severe clinical and histological risk factors, also considering the risk of poorly controllable infections. Hughley et al. reported five DEB patients who developed glomerulonephritis (5). All suffered from chronic SA cutaneous infections with similar renal pathology findings to that of our patient. Three of them were treated with steroids, MMF or angiotensin-converting enzyme inhibitor (ACEi). Their renal disease might be due to IAGN. While antibiotics contributed in part to their improvement, the correlation of antibiotic use with renal improvement did not prove causation. Additionally, a randomized controlled trial showed MMF emerged as a possible treatment option in DEB patients (19). However, it is critical to reduce infectious complications without compromising therapeutic efficacy.

In conclusion, we presented a rare case of DEB with anti-MPO and anti-PR3 seropositivity, accompanied by glomerular IgA deposits. It is uncertain whether this condition was an incidental finding or a novel clinical entity. We even propose that this case may be diagnosed with SAGN, or that SAGN may have superimposed on IgAN. Lastly, the patient showed efficient attenuation of hemato-proteinuria and ANCA turning negative with glucocorticoid, immunosuppressant, ACEi, and anti-infection treatment.

## Data Availability Statement

The datasets for this article are not publicly available due to concerns regarding participant/patient anonymity. Requests to access the datasets should be directed to the corresponding author.

## Ethics Statement

The studies involving human participants were reviewed and approved by the Children’s Hospital, Zhejiang University School of Medicine. Written informed consent to participate in this study was provided by the participants’ legal guardian/next of kin. Written informed consent was obtained from the minor(s)’ legal guardian/next of kin for the publication of any potentially identifiable images or data included in this article.

## Author Contributions

LY and GH drafted the manuscript. ZL was in charge to collect and interpret the data. WG and JL performed and interpreted histological investigations. JM and JW critically revised the manuscript. All authors contributed to manuscript revision and approved the submitted version.

## Conflict of Interest

The authors declare that the research was conducted in the absence of any commercial or financial relationships that could be construed as a potential conflict of interest.

## Publisher’s Note

All claims expressed in this article are solely those of the authors and do not necessarily represent those of their affiliated organizations, or those of the publisher, the editors and the reviewers. Any product that may be evaluated in this article, or claim that may be made by its manufacturer, is not guaranteed or endorsed by the publisher.
